# MiR-378 is an independent prognostic factor and inhibits cell growth and invasion in colorectal cancer

**DOI:** 10.1186/1471-2407-14-109

**Published:** 2014-02-20

**Authors:** Guang-jun Zhang, He Zhou, Hua-xu Xiao, Yu Li, Tong Zhou

**Affiliations:** 1 The First Department of General Surgery, The Affiliated Hospital of North Sichuan Medical College, Nanchong, Sichuan, People’s Republic of China; 2 Institute of Hepatobiliary, Pancreatic and Intestinal Disease, North Sichuan Medical College, Nanchong, Sichuan, People’s Republic of China; 3 Department of Pathology, The North Sichuan Medical College, Nanchong, Sichuan, People’s Republic of China; 4 Department of Microbiology and Parasitology, North Sichuan Medical College, Nanchong, Sichuan, People’s Republic of China

**Keywords:** Colorectal cancer, miR-378, Vimentin, Invasion, Prognosis

## Abstract

**Background:**

MicroRNAs(miRNAs) are small non-coding RNAs that participate in a variety of biologic processes, and dysregulation of miRNA is always associated with cancer development and progression. Aberrant expression of miR-378 has been found in some types of cancer. However, effects and potential mechanisms of miR-378 in colorectal cancer (CRC) have not been explored.

**Methods:**

Quantitative RT-PCR was performed to evaluate miR-378 levels in CRC cell lines and 84 pairs of CRC cancer and normal adjacent mucosa. Kaplan–Meier and Cox proportional regression analyses were utilized to determine the association of miR-378 expression with survival of patients. MTT and invasion assays were used to determine the role of miR-378 in regulation of CRC cancer cell growth and invasion, respectively. Tumor growth was assessed by subcutaneous inoculation of cells into BALB/c nude mice. Luciferase assay was performed to assess miR-378 binding to vimentin gene.

**Results:**

In this study, we confirmed that miR-378 significantly down-regulated in CRC cancer tissues and cell lines. Moreover, patients with low miR-378 expression had significantly poorer overall survival, and miR-378 expression was an independent prognostic factor in CRC. Over-expression of miR-378 inhibited SW620 cell growth and invasion, and resulted in down-regulation of vimentin expression. However, miR-378 knock-down promoted these processes and enhanced the expression of vimentin. In addition, we further identified vimentin as the functional downstream target of miR-378 by directly targeting the 3′-UTR of vimentin.

**Conclusions:**

In conclusion, miR-378 may function as a tumor suppressor and plays an important role in inhibiting tumor growth and invasion. Our present results implicate the potential effects of miR-378 on prognosis and treatment of CRC cancer.

## Background

Colorectal cancer (CRC) is one of the most common malignancies worldwide, and it has high mortality and prevalence rates in East Asian countries including China [1]. Metastasis is the major cause of CRC morbidity and mortality, and more than one-third of patients with CRC will ultimately develop metastatic disease [2]. An urgent need to search for specific, sensitive biomarkers for the early diagnosis and prognosis prediction of CRC exists.

miRNAs are a class of small non-coding RNAs, which contain of about 22 nucleotides. miRNAs bind to partially complementary sequences in the 3`-untranslated region (UTR) of specific target mRNA, resulting in either mRNA degradation or translation inhibition [3]. Growing evidence suggests that miRNAs play an important role in various biologic processes, including cell proliferation, development, and differentiation [4,5]. Furthermore, increasing numbers of miRNAs have been observed in various types of cancer and may be involved in modulating cancer cell behaviors [6-9]. These data emphasize the importance of miRNAs in cancer development and provide new insights into understanding the molecular mechanism of tumorigenesis.

Alterations in miRNA expression have been suggested to play important roles in tumorigenesis and cancer progression [10]. Recently, miR-378 expression was shown to be deregulated in oral carcinoma and renal cell carcinoma [11,12]. The involvement of miR-378 in the tumorigenesis and metastasis of glioblastoma, non-small cell lung cancer, breast cancer and gastric cancer has also been reported [13-16]. Previous reports revealed that the miR-378 was down-regulated in CRC [17-20]. However, to our knowledge, its biological role and clinical significance in colorectal cancer remain undefined.

Therefore, in this study, we confirmed the expression of miR-378 in fresh CRC tissue specimens and CRC cell lines by using qRT-PCR. After that, we assessed the clinical significance of miR-378 in colorectal cancer, and to investigate the effects of miR-378 on CRC cells growth and invasion and further discuss the mechanisms of action of miR-378 by identifying its potential target gene.

## Methods

### Patients and tissue samples

Surgical specimens of cancer tissue and adjacent normal mucosa were obtained from 86 patients with colorectal cancer who underwent surgery at The Affiliated Hospital of North Sichuan Medical College between 2005 and 2008. Among the 86 patients, 2 patients diagnosed with distant metastases were excluded from our study because these cases were too few for meaningful statistical analysis. None of the patients had received chemotherapy or radiotherapy before surgery excision. After collection, all tissue samples were immediately frozen in liquid nitrogen and stored at −80°C until use. Tumor stage was classified according to the International Union against Cancer (UICC, 6th ed., 2002). Informed written consent was obtained from each patient, and research protocols were approved by the Medical Ethics Committee of North Sichuan Medical College.

### Cell culture

The human CRC cell lines HT29, HCT116, SW480, SW620 and the normal colon epithelium cell line CCD-18Co were obtained from the American Type Culture Collection and cultured in DMEM medium supplemented with 10% fetal bovine serum, 100u/ml penicillin and 100 mg/ml streptomycin, at 37°C in a humidified atmosphere of 5% CO_2_.

### RNA extraction and real-time RT–PCR

Total RNA was extracted using TRIzol reagent (Invitrogen, Carlsbad, CA, USA). The PCR primers for miR-378 and U6 were purchased from Applied Biosystems (ABI, Foster City, CA, USA). The PCR primers for vimentin were 5′-GAGAACTTTGCCGTTGAAGC-3′ and 5′-GCTTCC TGTAGGTGGCAATC-3′. The primers for β-actin: 5′-CCAAGGCCAAC CGCGAGAAGATGAC-3′ and 5′-AGGGTACATGGTGGTGCCGCCA GAC-3′. The first-strand cDNA was synthesized using the PrimeScript RT reagent Kit (TaKaRa, Dalian, China). Real-time PCR was performed using SYBR Premix Ex Taq (TaKaRa) and measured in a LightCycler 480 system (Roche, Basel, Switzerland). U6 or β-actin was used as internal control. Relative quantification of microRNA expression was calculated using the 2^-ΔΔCT^ method.

### Transfection of miRNA

The pre-miR miRNA-378(Pre-miR-378), pre-miR negative control (Pre- miR-nc), anti-miR negative control (anti-miR-nc) and anti-miR-378 inhibitor (anti-miR-378) were purchased from Ambion (Austin, TX,USA). 2 × 10^5^ cells were seeded into each well of a 6-well plate and transfected for 24 h or 48 h using Lipofectamine 2000 reagent (Invitrogen) following manufacturer’s protocol. Transfected cells were used in further assays or RNA/protein extraction.

### MTT assay

A total of 2 × 10^4^ SW620 cells were plated onto 96-well plates for 24 h. The cells were then transfected with 50 nM the indicated miRNA. At different time points (24 h, 48 h and 72 h), the culture medium was removed and replaced with culture medium containing 10 μl of sterile MTT dye (5 mg/ml). After incubation at 37°C for 4 h, the MTT solution was removed, and 150 μl dimethyl sulfoxide (DMSO) was added to each well followed by measuring the absorbance at 570 nm on an enzyme immunoassay analyzer (Bio-Rad, Hercules, CA, USA).

### Matrigel invasion assay

Cell invasion experiment was assessed using the Matrigel Invasion Chamber of pore size 8 mm (Corning Costar Corporation, Cambridge, MA, USA). A total of 5 × 10^4^ cells were seeded into the upper compartment of the chamber coated with 150 μg of Matrigel (BD Biosciences, Bedford, MD,USA). Medium containing 10% fetal bovine serum in the lower chamber served as the chemoattractant. After the cells were incubated for 48 hours and fixed and stained with hematoxylin for 30 minutes, and the non-invaded cells were removed with cotton swabs. The number of invasive cells on the lower surface of the membrane was then counted under a microscope at a magnification of × 400 in 5 random fields.

### In vivo xenograft experiments

Female BALB/C nude mice at the age of 4 weeks purchased from the Shanghai Laboratory Animal Center (Chinese Academy of Sciences) were randomly divided into 2 groups (five mice per group). All the procedures involving animals were approved by Experimental Animal Ethics Committee, North Sichuan Medical College. Pre-miR-378 or pre-miR-nc stable transfection SW620 cells suspensions (1 × 10^6^ cells/ml) in 200 μl serum-free medium were subcutaneously injected into the flanks of nude mice, respectively. Tumor growth was examined twice per week for 5 weeks and tumor volumes were calculated using the formula Volume (mm^3^) = L × W^2^ /2 (length L, mm; width W, mm). After 5 weeks, tumor samples were carefully removed and weighed.

### Western blot analysis

Immunoblotting was performed to detect the expression of vimentin in CRC cell lines. Transfected cells were lysed in RIPA lysis buffer (ProMab Biotechnology). Protein was loaded onto a SDS-PAGE minigel and transferred onto PVDF membrane. After probed with 1:500 diluted mouse anti-vimentin (Santa Cruz Biotechnology, Santa Cruz, CA, USA) at 4°C overnight, the blots were subsequently incubated with HRP-conjugated secondary antibody (1:5000). Signals were visualized using ECL Substrates (Millipore, MA, USA). GAPDH was used as an endogenous protein for normalization.

### Dual-luciferase reporter assay

For luciferase reporter experiments, the wild-type and mutated 3′UTR of vimentin mRNA were subcloned into the *Xho*I and *Not*I site of the psicheck-2 vector (Promega) and the new vectors were named psicheck-2-vimentin-WT and psicheck-2-vimentin-MUT, respectively. The following primers were used to amplify specific fragments: vimentin-WT, forward 5′- CACAACTCGAGTTGCACACACTCAGTG CAGC-3′ and reverse 5′- AAGGAAAAAAGCGGCCGCCAAGTTGG TTGGATACTTGCTGG-3′and vimentin-MUT, forward 5′- GTTTTAG TCCTGCGCAAGATAGATTTGGAATAGGA-3′and Reverse 5′- TGC GCAGGACTAAAACTGCAGAAAGGCACTTGAAAGCTG-3′. For reporter assay, HEK 293 T cells were plated onto 24-well plates at 2 × 10^4^ cells/well and transfected with 200 ng of psicheck-2-wimentin-WT or psicheck-2-vimentin-MUT and 40 nM pre-miR-378 or pre-miR-nc using Lipofectamine 2000 (Invitrogen). Firefly luciferase was used to normalize the Renilla luciferase. After transfection for 48 h, cells were harvested and assayed with Dual-Luciferase Reporter Assay System (Promega) according to the manufacturer’s protocols.

### Statistical analysis

All data presented in this study have been repeated at least three times from three independent experiments. Continuous variables were expressed as the mean ± standard deviation. The differences between groups were analyzed using student’s *t*-test, while categorical data were studied using chi-square test. The postoperative survival rate was analyzed with Kaplan–Meier method, and differences in survival rates were assessed with log-rank test. A Cox proportional hazards model was used for multivariate analysis. All statistical analyses were performed using SPSS 16.0 software (SPSS, Chicago, IL, USA). Two-sided *P*-values were calculated, and differences were considered significant at *P*-values of <0.05.

## Results

### The miR-378 expression in CRC tissues and cell lines

**Figure 1:**
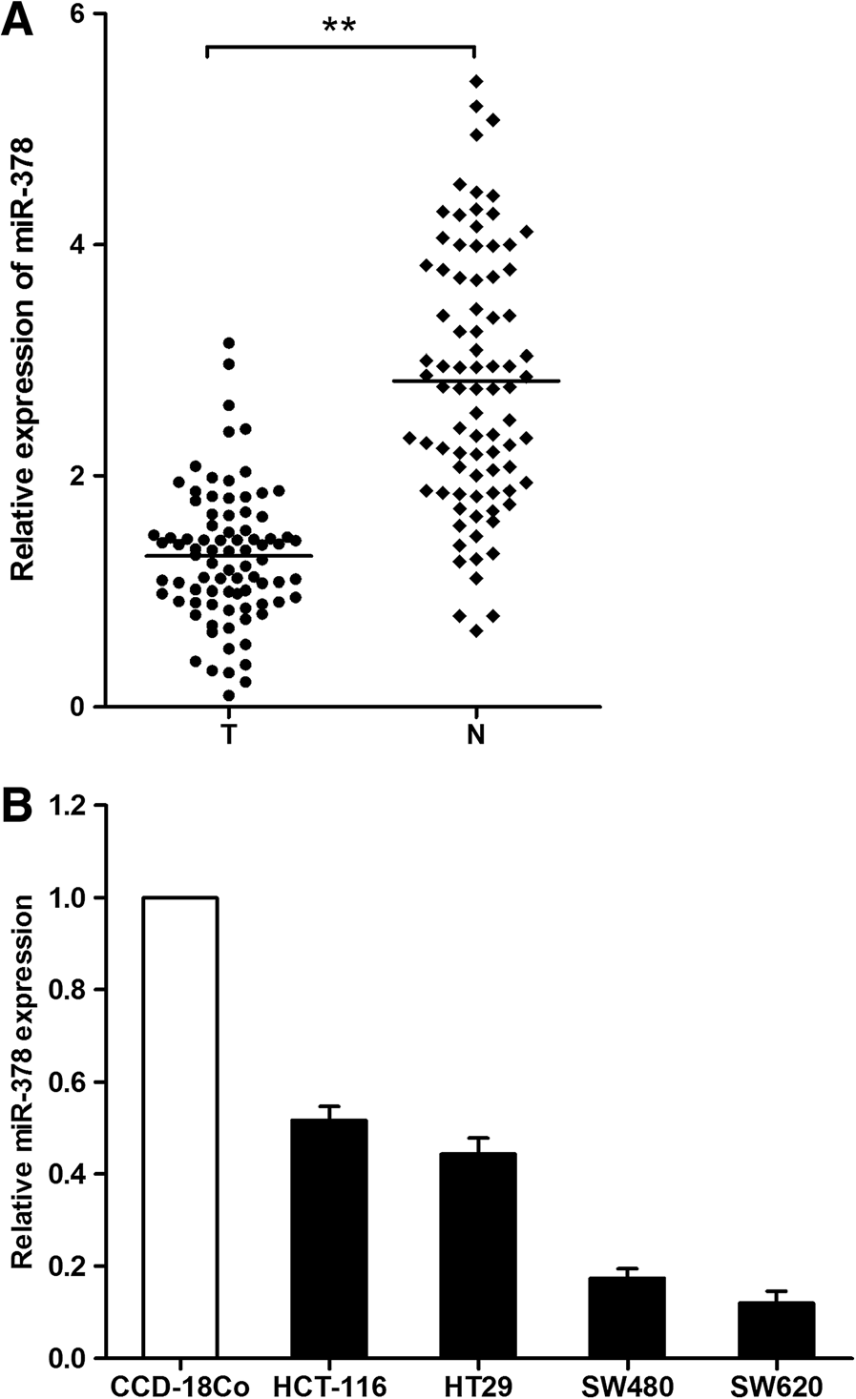
**The relative expression levels of miR-378 in CRC tissues and cell lines. (A)** The relative expression of miR-378 in CRC tissues (T) and adjacent normal mucosa (N). The bars in the figure indicate the means of the relative expressions of miR-378. **(B)** The relative expression of miR-378 in CRC cell lines SW620, SW480, HCT116, HT29 and the normal colon epithelium cell line CCD-18Co. **P < 0.01.

We performed quantitative PCR analysis to detect the expression level of miR-378 in CRC tissues and cell lines. In 84 cases of primary CRC tissues and their adjacent normal colonic tissues, our results showed that miR-378 was significantly decreased in CRC tissues (1.31 ± 0.57) when compared with that in the paired adjacent normal tissues(2.82 ± 1.12) (*P* < 0.01, Figure 1A). The expression of miR-378 was then assessed in four CRC cell lines (SW620, SW480, HT29 and HCT116) and the normal colon epithelium cell line CCD-18Co. The results showed that the level of miR-378 was lower in all four CRC cell lines compared with normal colon cell line (Figure 1B).

### Correlation between miR-378 expression and clinical features and prognosis of CRC patients

To evaluate the correlation between miR-378 expression and clinicopathological characteristics, the 84 patients with CRC cancer were classified into two groups according to the median expression (2.77) of miR-378. We compared the clinicopathological factors of the high and low miR-378 expression group (Table 1) and found that low expression of miR-378 was significantly correlated with large tumor size (*P* = 0.035), positive lymph node metastasis (*P* = 0.004), and advanced clinical stage (*P* = 0.017).

**Table 1:** Association of miR-378 expression with clinicopathologic factors of colorectal cancer patients

**miR-378 expression**			
**Variable**	**Low (n = 42)**	**High (n = 42)**	***P-value***
Age (years)			0.268
≤ 60	15	20	
> 60	17	22	
Gender			0.378
Male	26	22	
Female	16	20	
Tumor size			0.035
≤ 5 cm	24	33	
> 5 cm	18	9	
Histological grading			0.172
Well, moderate	24	30	
Poor, mucinous	18	12	
Depth of invasion			0.275
T1-T2	19	24	
T3-T4	23	18	
Clinicopathological staging			0.017
I	4	14	
II	9	12	
III	29	16	
Location			0.355
Colon	12	16	
Rectum	30	26	
Lymph node metastasis			0.004
Negative	13	26	
Positive	29	16	

well: well-differentiated, moderate: moderately differentiated, poor: poorly differentiated, mucinous: mucinous carcinoma.

**Figure 2:**
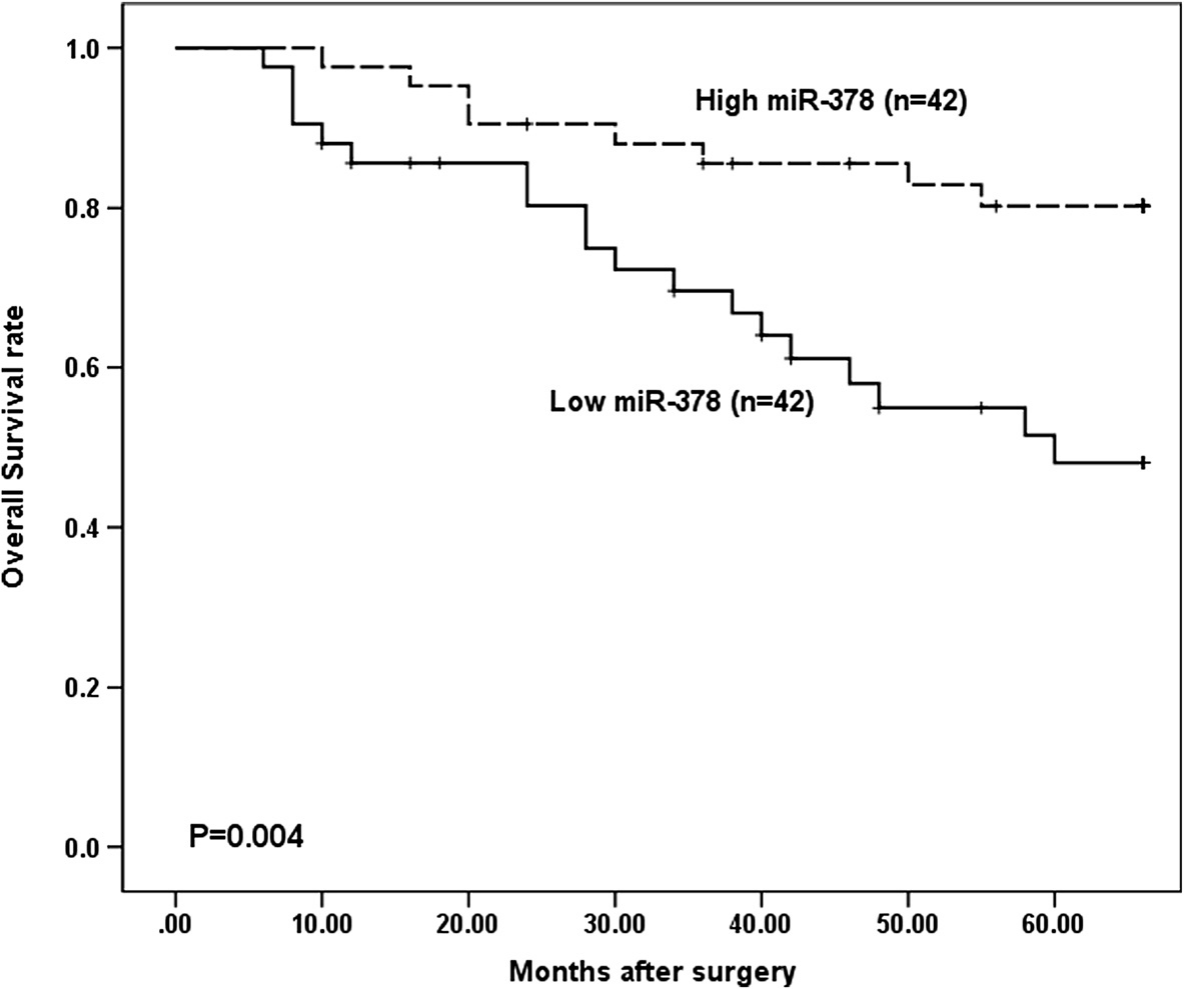
**Kaplan–Meier survival curves of patients with colorectal cancer based on miR-378 expression status.** Patients in the low expression group had significantly poorer prognosis than those in high expression group (*P* = 0.004, log-rank test).

Kaplan–Meier survival analysis showed that low miR-378 expression correlated with shorter overall survival (Figure 2, *P* = 0.004). Univariate proportional hazard model revealed a statistically significant correlation between overall survival and miR-378 level, tumour size, local invasion, lymph node metastasis, and TNM stage (Table 2). The parameters that significantly correlated with survival in the univariate analysis were further assessed by multivariate analysis. Since TNM stage is determined by the local invasion and lymph node metastasis, it was not further enrolled into the multivariate analysis in this study, and the results of the multivariate analysis demonstrated that miR-378 (*P* = 0.037) and lymph node metastasis (*P* = 0.001) were independent prognostic factors for overall survival (Table 2). These results indicated that miR-378 may be involved in the progression of CRC and predict overall survival in CRC.

**Table 2:** Univariate and multivariate analyses of prognostic factors in colorectal cancer

**Variable**	**Univariate analysis**			**Multivariate analysis**		
	**HR**	**95% CI**	***p*-value**	**HR**	**95% CI**	***P*-Value**
Age (years)	1.619	0.759-3.454	0.213			
Gender	1.121	0.521-2.422	0.766			
Location	1.691	0.783-3.652	0.181			
Histological grading	1.844	0.861-3.949	0.115			
Tumor size	2.414	1.123-5.186	0.024	2.086	0.954-4.563	0.066
Depth of invasion	2.374	1.083-5.202	0.031	1.461	0.650-3.282	0.358
Lymph node metastasis	5.286	1.996-13.999	0.001	5.080	1.940-13.554	0.001
Clinicopathological staging	2.863	1.268-6.564	0.013			
MiR-378	3.165	1.381-7.253	0.006	2.492	1.057-5.876	0.037

HR: hazard ratio, CI: confidence interval.

### Effect of miR-378 on CRC cell growth and invasion in vitro

**Figure 3:**
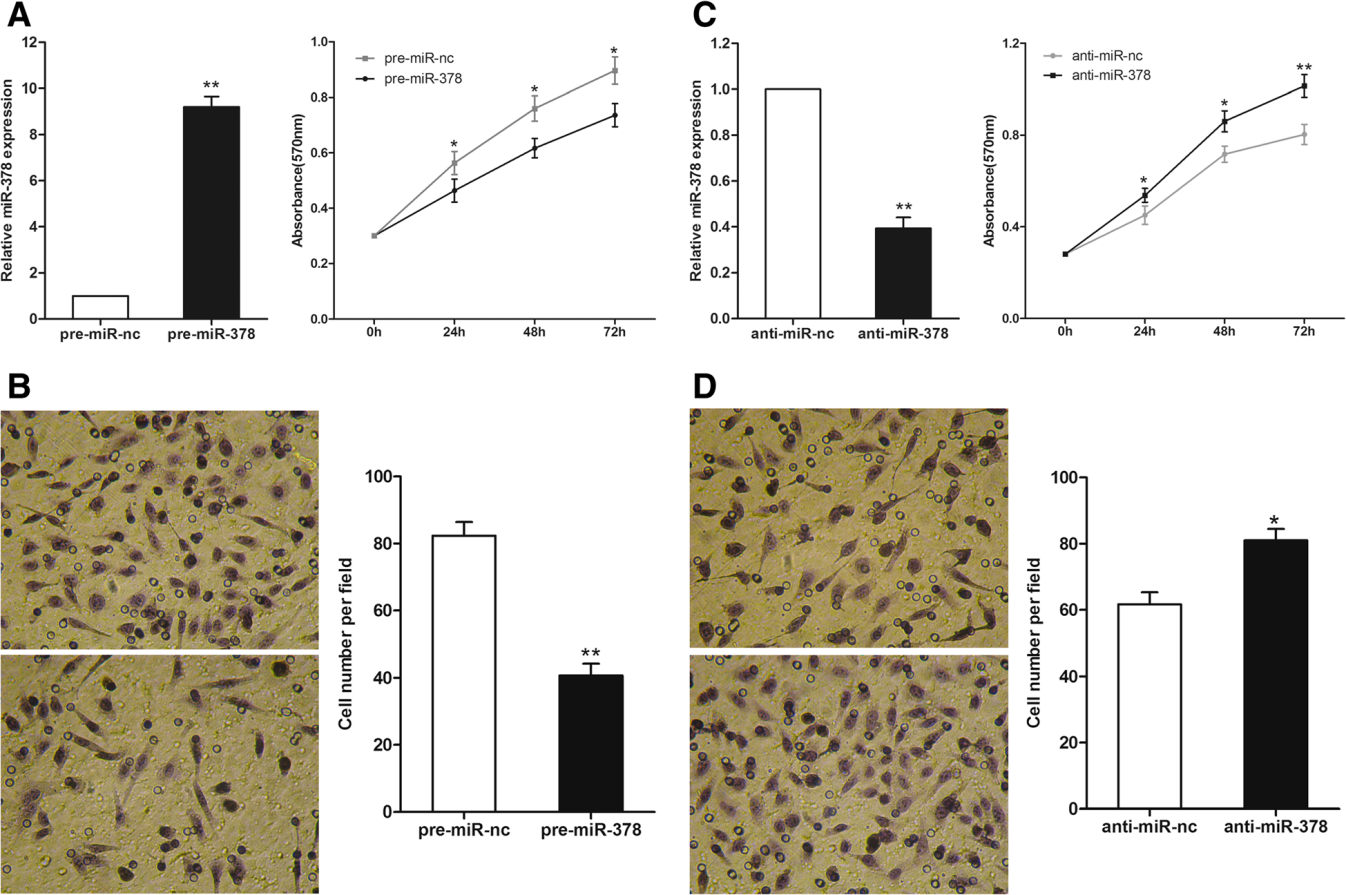
**Effects of miR-378 on proliferation and invasion of SW620 cell line. (A) (B)** Over-expression of miR-378 inhibited SW620 cell growth and invasion. **(C) (D)** Down-regulation of miR-378 promoted SW620 cell growth and invasion. *P < 0.05, **P < 0.01.

To validate if miR-378 regulates CRC cell growth, we performed a proliferation assay by transfecting pre-miR-378 or pre-miR-nc into SW620 cells. It showed that the increased expression of miR-378 induced significant inhibition on cell proliferation (Figure 3A). Correspondingly, after transfected with anti-miR-378, SW620 cells presented stimulated cell growth compared to scramble control (Figure 3C). Cell motility of transfected cells was evaluated by invasion assay. As shown in Figure 3B, compared to the scramble control, pre-miR-378 transfected SW620 cells exhibited significant impairment of invasive ability. Inversely, down-regulation of miR-378 in inhibitors transfected SW620 cells apparently promoted cell invasion (Figure 3D).

### MiR-378 inhibits tumor growth in vivo

**Figure 4:**
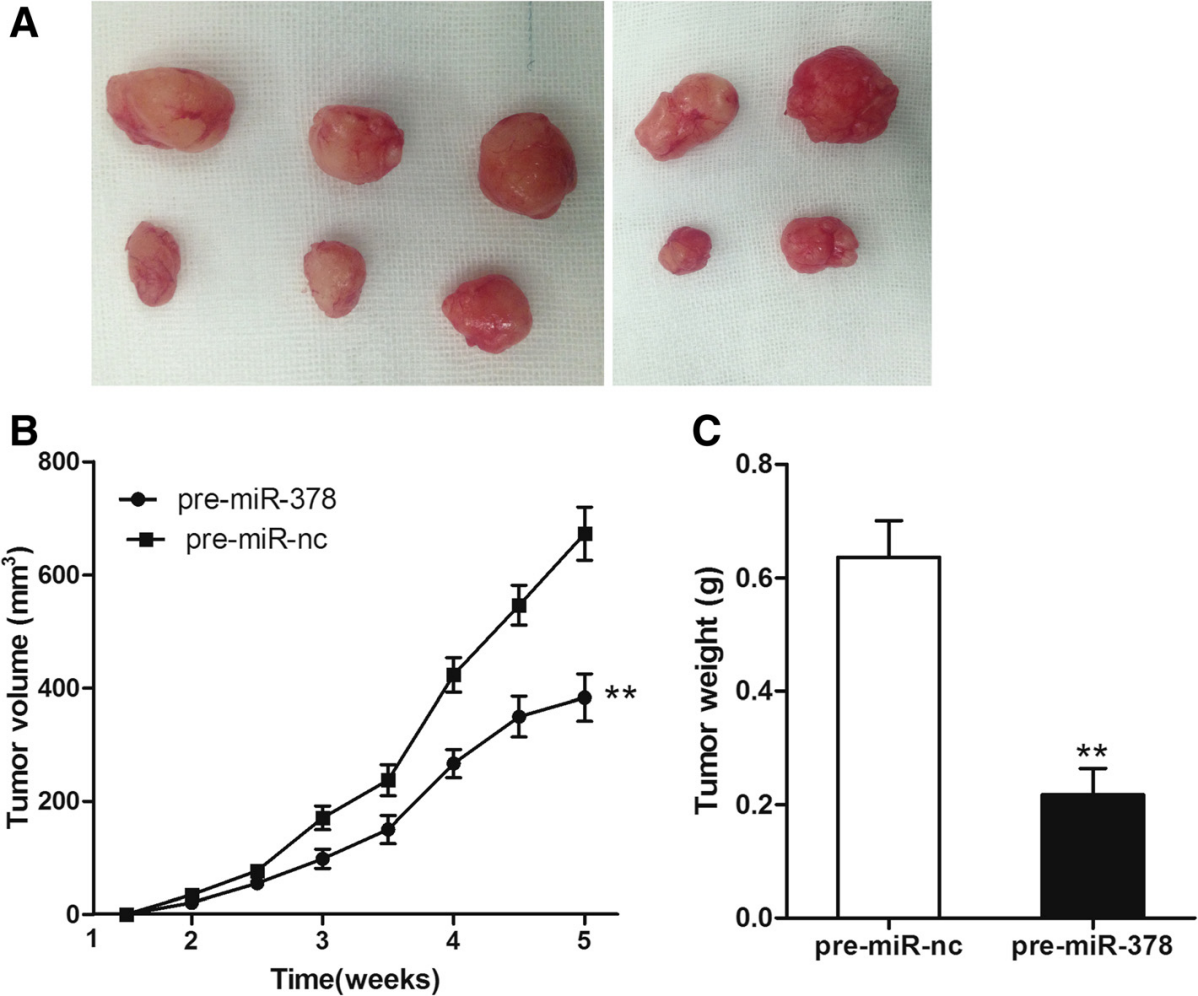
**MiR-378 inhibits tumor growth in vivo. (A)** Representative image of tumors formed. **(B)** Growth curve drawn by measuring tumor volumes at the indicated times. **(C)** Weight of xenograft tumors. **P < 0.01.

To examine the role of miR-378 in CRC tumor development, we used a BALB/C nude xenograft mouse model in which mice were transplanted with pre-miR-378 and pre-miR-nc transfected cells. After 5 weeks, miR-378 over-expressing tumors were significantly smaller than those of mice transfected with scramble control (Figure 4A). Furthermore, overexpression of miR-378 significantly reduced xenograft tumor volume (Figure 4B) and tumor weight (Figure 4C).

### Vimentin is a direct target of miR-378

**Figure 5:**
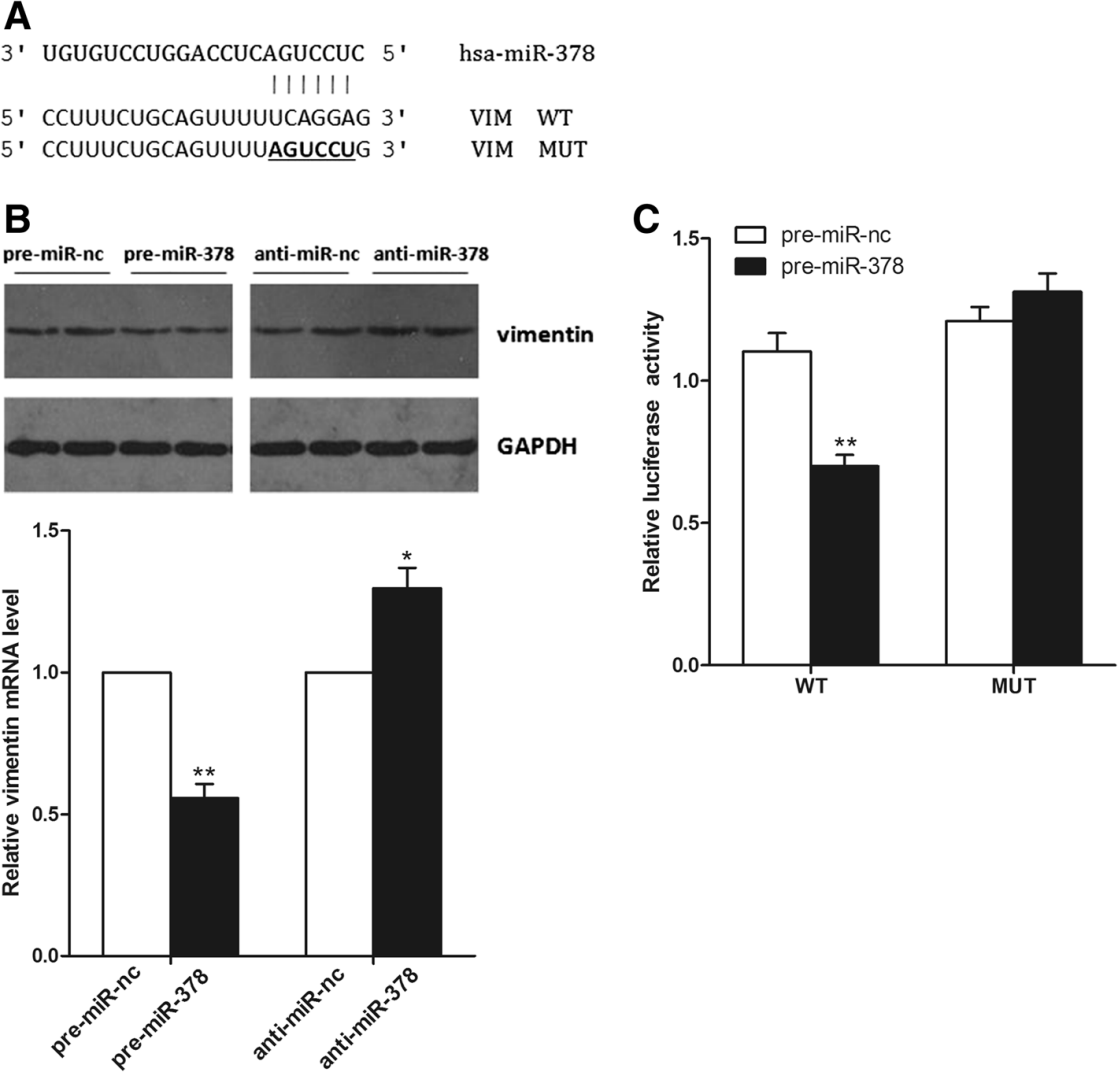
**Vimentin is a direct target of miR-378. (A)** The wild-type (WT) and mutated (MUT) 3′UTR of vimentin, with the seed region and base substitutions (bold). **(B)** The expression levels of vimentin mRNA and protein were detected by qRT-PCR and western blot assays. **(C)** Ectopic miR-378 expression inhibits wild-type but not mutant vimentin 3′UTR reporter activity. *P < 0.05, **P < 0.01.

To understand the possible mechanisms that might underlie miR-378-mediated growth and metastasis suppression, we performed in silico studies to search for potential gene targets of miR-378 using the bioinformatics algorithms Pictar and miRanda. All of the algorithms indicated that vimentin was a theoretical target of miR-378 (Figure 5A).

To further confirm that vimentin is the direct target of miR-378, we first determined whether over-expression of miR-378 can lead to down-regulation of vimentin expression. We transfected SW620 cells with pre-miR-378 or anti-miR-378, Western blot showed that the enhanced miR-378 expression in SW620 cells significantly repressed vimentin protein expression compared to cells transfected with scramble control (Figure 5B). Relatively, down-regulation of miR-378 by inhibitors in SW620 cells led to a moderate increase of vimenin protein level (Figure 5B). Meanwhile, apparent alterations of vimentin mRNA expression were also observed by quantitative PCR (*P* < 0.01, Figure 5B). It suggested a potential regulation of vimentin by miR-378. Thus, vimentin is likely to be suppressed by miR-378 through translational inhibition and mRNA degradation.

To directly address whether miR-378 binds to the 3′-UTR of target mRNA, we generated a luciferase reporter vector that contain the vimentin 3′-UTR with the putative miR-378 binding sites. Correspondingly, we also generated a mutant reporter vector which contains the vimentin 3′-UTR with a mutation at the putative miR-378 binding site (Figure 5A). As shown in Figure 5C, we observed a marked reduction in luciferase activity in cells transfected with pre-miR-378 compared with pre-miR-nc transfected cells (*P* < 0.01). In contrast, no change of luciferase was observed in cells transfected with the mutant 3′-UTR constructs. Taken together, these findings indicate that vimentin is a direct, downstream target for miR-378 in SW620 cells.

## Discussion

Several groups have screened for colon cancer-related miRNAs, but limited evidence exists that causally link specific miRNAs to specific functions [21]. Identification of cancer-specific miRNAs and their targets is critical for understanding their roles in tumorigenesis, and may be important for finding out novel prognostic and therapeutic targets.

Accumulating evidence showed that up-regulation of miR-378 was associated with several types of human malignant solid tumors, including those of the glioblastoma, breast cancer and renal cell carcinoma [12,13,15]. In these types of cancer, miR-378 seemed to be an oncogene, and enhanced tumor cell survival, promoted tumor growth and metastasis in some tumors via regulation of the target genes SuFu, Fus-1, HMOX1, ESRRG and GABPA [12-15]. However, other studies demonstrated that miR-378 was down-regulated in gastric cancer and oral cancer [11,16], and miR-378 may act as tumor suppressors in gastric cancer by negatively regulating the expression of CDK6 and VEGF [16]. Therefore, the function of miR-378 is complicated because it can be oncogenic or a tumor suppressor in different types of cancers. Several studies have been reported that miR-378 was significantly down-regulated in CRC [17-20]. However, the specific mechanism by which the altered expression of miR-378 affects tumor development and progression has not been elucidated and its involvement in CRC has not been addressed in detail.

In the current study, we further confirmed that miR-378 expression was significantly down-regulated in CRC tissue samples and cell lines, and that loss of miR-378 expression was associated with large tumor size, advanced clinical stage, lymph node metastasis and shorter overall survival of the patients with CRC, indicating that miR-378 might be involved in CRC progression and could be used as a potential prognostic biomarker in CRC. Furthermore, over-expression of miR-378 could significantly inhibit cell proliferation and invasion in vitro and tumor growth in vivo. On the contrary, when transfected with miR-378 inhibitors, SW620 cells exhibited stimulated proliferation as well as invasive capabilities. This study first showed that miR-378 may function as a tumor suppressor in CRC.

To explore the mechanisms underlying the inhibition of CRC cell growth and invasion mediated by miR-378, we next set out to identify the potential target genes of miR-378. The bioinformatics analysis indicates that vimentin may be the potential target for miR-378. One of the key molecular steps in the process of distant metastasis includes epithelial-to-mesenchymal transition (EMT) [22], which permits invasion and migration in CRC [23], and is associated with a poor prognosis in CRC [24]. The intermediate filament protein (IFP) vimentin, expressed in mesenchymal cells, is a well-known marker for EMT [25]. Vimentin expression and perturbation of E-cadherin-mediated cell adhesion are therefore both regarded as hallmarks of EMT-associated events [26]. A recent report showed that vimentin was one of the predominant overexpressed proteins in the highly metastatic cell line SW620 [27]. Thus, in the present study, SW620 cells were selected as model systems for the study of the molecular events involved in CRC metastasis. A previous study suggested that vimentin methylation was associated with liver metastasis and peritoneal dissemination in colorectal cancer [28]. Vimentin was also reported to be highly expressed in CRC, and high expression of vimentin was found to be associated with lymph node metastasis and disease recurrence in CRC [29,30].

Several pieces of evidence in our study indicate that vimentin is a direct target gene of miR-378 in CRC cancer. Firstly, at both mRNA and protein level, up-regulation of miR-378 expression in SW620 cells effectively suppressed vimentin expression, whereas, downregulation of miR-378 moderately promoted vimentin expression. It suggested a potential inverse relevance of miR-378 and vimentin in CRC. Secondly, over-expression of miR-378 significantly reduces the activity of a luciferase reporter containing the 3′UTR sequence of vimentin. In addition, vimentin has been confirmed as a target gene of miR-378 in glioblastoma Cells [30]. When combined with bioinformatic analysis, we concluded vimentin was a target gene of miR-378 in CRC. Taken together, these findings sufficiently consolidated that miR-378 played a suppressive role in cellular proliferation and invasion, at least, in part due to directly inhibiting vimentin expression.

## Conclusion

In summary, our present study showed that miR-378 was down- regulated in CRC tissues and cell lines. And the low expression pattern was observed to be significantly correlated with increased tumor size, advanced clinical stage, lymph node metastasis and worse prognosis. We also found that miR-378 can inhibit tumor growth and invasion partly by targeting vimentin. Our data implicated the potential application of miR-378 as a tumor suppressor in CRC therapy and also as a tumor marker for predicting prognosis.

## Competing interests

The authors declare that they have no competing interests.

## Authors’ contributions

ZGJ, XHX and LY performed experiments; ZGJ, ZH and ZT designed research and wrote the paper; ZGJ and ZH analyzed data. All authors read and approved the final manuscript.

## Pre-publication history

The pre-publication history for this paper can be accessed here:

http://www.biomedcentral.com/1471-2407/14/109/prepub
